# An In Vivo Study on the Development of Bacterial Microbiome on Clear Orthodontic Retainer

**DOI:** 10.3390/dj10120239

**Published:** 2022-12-16

**Authors:** Kabilan Velliyagounder, Anil Ardeshna, Serena Shah

**Affiliations:** 1 Department of Oral Biology, Rutgers School of Dental Medicine, Newark, NJ 07103, USA; 2 Department of Orthodontics, Rutgers School of Dental Medicine, Newark, NJ 07103, USA; 3 Department of Periodontics, Rutgers School of Dental Medicine, Newark, NJ 07103, USA

**Keywords:** orthodontic appliance, Essix C+ orthodontic retainer, 16S rRNA gene sequencing, microbial community, orthodontic material

## Abstract

Objectives: The objective of this study was to see how the bacterial composition changes on clear orthodontic retainer over a 14-day period. Methods: Saliva and plaque samples collected from a clear retainer surface were obtained from five healthy volunteers receiving retainer treatment. Prior to clear retainer delivery, patients had not been wearing any other appliances. Patients were instructed to wear their clear retainer for the 14-day period, taking them off to eat and to clean them with a soft-bristle toothbrush. The bacterial composition was determined via Illumina MiSeq sequencing of the bacterial 16S rRNA. After bioinformatics processing using the QIIME pipeline, the intra- and intergroup biodiversity of the sample was analyzed. Results: The bacterial composition changed over a 14-day period in the saliva and on the clear retainer. When comparing the different phylum levels between saliva and clear retainer’ microbiota, the *Firmicutes* were significantly increased 1.26-fold (*p* = 0.0194) and 1.34-fold (*p* = 0.0123) after 7 and 14 days of retainer treatment when compared to saliva, respectively. The *Campylobacteriota* were significantly decreased 1.80-fold (*p* = 0.05) in the clear retainer when compared to saliva at 7 days. At the genus level, several microbiota were significantly increased in relative abundance in the clear retainer after the 14-day period. Conclusion: These findings reveal that the presence of a clear retainer in the mouth might lead to enamel changes or periodontal tissue destruction, especially after 14 days of use.

## 1. Introduction

The oral microflora is highly diverse and consists of over 700 bacterial species. Each individual has a “core” microbiome consisting of healthy bacteria that is common to all individuals, and a “variable” microbiome that consists of healthy and sometimes pathogenic bacteria [1]. Studies have shown that patients that are undergoing orthodontic treatment are subject to a greater risk of caries and gingival inflammation. Since it is hard for these patients to maintain proper oral hygiene, plaque and bacteria can accumulate on their teeth and around the gingival sulcus. The normal oral microflora consists of healthy bacteria, and when this accumulation occurs around the teeth and gingival sulcus, this can shift those bacteria from a healthy state to one that is able to cause continued destruction of the periodontium and increase the caries risk for the patient [2]. Understanding biofilms is of great importance, since nearly 90% of all microbes are associated with biofilm formation [3]. 

Studies have shown that environmental changes such as placing orthodontic brackets or wearing orthodontic appliances may shift the bacterial community from a healthy one to one that can cause disease [4]. Orthodontic treatment can cause an increase in biofilm accumulation due to the retention of bacteria on orthodontic appliances. This increased accumulation and poor oral hygiene can lead to increased caries, periodontal disease, and enamel decalcification. Common pathogenic bacteria in the oral cavity include *Streptococcus mutans*, *Porphyromonas gingivalis*, *Treponema denticola*, *Tannerella forsythia*, *Fusobacterium nucleatum* and *Aggregatibacter actinomycetemcomitans*. *S. mutans* has been found in areas associated with orthodontic appliances. It has been found to aid in caries development and enamel decalcification, and has been seen in areas of white-spot lesions and gingivitis post orthodontics [5,6,7] and gingivitis [8,9]. A systematic review found that the levels of these bacteria increased during orthodontic treatment, and three months after removal there was a significant reduction in the number of these pathogens [10]. The frequency of subgingival pathogenic bacteria was found to increase one month after the beginning of orthodontic treatment, and stayed consistently high for up to three months after the removal of orthodontic appliances [11]. 

Studies have been conducted showing bacterial adhesion to orthodontic brackets. A study by Pellissari et al. collected biofilm samples around fixed brackets, fixed straight arch wires and fixed modular elastomeric ties in orthodontic patients. They found that patients not wearing fixed orthodontic appliances harbored microorganisms that are commonly found in a healthy oral cavity and those that are associated with enamel. The prevalence of pathogenic Gram-negative microorganisms was higher in the oral cavity of patients wearing orthodontic appliances [12]. Another study by Papaioannou et al. looked at bacterial adherence to stainless steel, ceramic and plastic brackets, and found that the type of bracket did not affect the adherence of bacteria to the bracket [13]. The frequency of subgingival pathogenic bacteria was found to increase one month after the beginning of orthodontic treatment and stayed consistently high for up to three months after the removal of orthodontic appliances [11]. Previous studies have shown that when stainless steel brackets are placed, there is an increase in the biofilm found on the tooth and in periodontal colonizers [14].

In recent years, aligner therapy for orthodontics has become increasingly popular. They are an aesthetic and comfortable alternative to fixed orthodontics, and facilitate improved oral hygiene. Invisalign, a popular aligner brand, gives a series of removable trays to patients to use up to two weeks each [15]. A recent study found that from 0 to 24 h, the abundance of bacteria in the genera *Firmicutes*, *Lactobacillales*, *Bacteroides*, *Granulicatella*, *Porphyromonas*, *Prevotella*, *Haemophilus*, *Acinetobacter* and *Streptococcus* increased significantly on uncleaned retainers [16]. It has been reported that there were increased levels of *Campylobacter rectus*, *F. nucleatum*, *Prevotella melaninogencia*, *Prevotella intermedia*, *Fusobacterium periodontium*, *T. denticola*, *T. forsynthia* and *P. gingivalis* when the clear aligner was used as a treatment therapy [17]. Another study found increased levels of *S. mutans* in patients treated with removable appliances compared to untreated groups [18]. Guo et al. found that *Firmicutes*, *Actinobacteria,* and *Tenericutes* were more abundant during the course of aligner therapy [19]. A recent (2022) study found that the amounts of *Streptococcus* and *Granulicatella* were significantly elevated on clear aligner trays compared with tooth-associated plaque communities. They found, however, that *Actinomyces*, *Corynebacterium* and *Selenomas* decreased on the clear aligner throughout treatment, indicating that the clear aligner tray becomes colonized by different bacteria than the plaque community, since the tray can act as a reservoir for pathogenic bacteria [20]. 

Many studies discuss biofilm adherence on conventional fixed orthodontic appliances, and the comparison of biofilm adherence on conventional versus removable appliances for orthodontics. There are also many studies looking at the biofilm composition on traditional orthodontic appliances. There are limited studies that look at the bacterial composition on clear aligners and retainers and in saliva, and how this changes over time. One study looked at the change in bacterial composition over a 24 h period, but to our knowledge, this is the only study that looks at the change in bacterial composition at 0, 7 and 14 days on both a clear retainer and in the saliva. Essix C+ is a popular thermoplastic material used for fabrication of clear aligners and retainers. An aligner is an active appliance for moving teeth, whereas a retainer is a passive appliance for holding teeth in that position. Therefore, we wanted to investigate the plaque that accumulates on orthodontic clear retainers and salivary bacteria in patients wearing clear retainers for a 14-day period. Based on previous and our own studies, we hypothesize changes in the composition of the plaque formed on the clear retainer and salivary bacteria from day 7 to day 14. In this in vivo prospective clinical study, we investigate the shift in bacterial composition on orthodontic clear retainers and in the saliva over a 14-day period using the 16S rRNA sequence. 

## 2. Materials and Methods

This is a pilot study conducted as a prospective clinical study involving the examination of orthodontic clear retainers at different time points in human subjects. Five adults were recruited and written informed consents were obtained from each participant before the performance of the study. Ethical approval for the study was granted by the IRB, Rutgers University (IRB protocol # Pro2018001627). The following inclusion criteria were applied: well controlled periodontal patients being treated in the Rutgers School of Dental Medicine Periodontics department, subjects aged 25–65, male or female, any ethnicity with a complement of all permanent teeth from first molar to first molar. Exclusion criteria consisted of patients with poor systemic health, previously wearing retainers prior to the study, tobacco use, professional cleaning or scaling completed within two weeks prior to the study, documented poor oral hygiene, anyone requiring dental prophylaxis, heart murmur, history of rheumatic fever, currently pregnant patients, patients currently on antibiotics or having taken antibiotics 1 month prior to participating in the study, or patients on chronic NSAIDs or chronic steroids. On day 1 (T0) the patients were assessed as to whether they meet the inclusion criteria by undergoing a periodontal examination and answering questions about their oral hygiene and health history. Patients then received prophylaxis and oral hygiene instructions, including receiving a free toothbrush and toothpaste, which they were instructed to use for the remainder of the study. Oral hygiene instructions included a demonstration and instructions on brushing twice a day and flossing once a day. Subjects were instructed not to use any other oral hygiene products for 14 days. Subjects were advised not to brush or floss on the morning of sample collection. Subjects were instructed to wear the clear retainer throughout the 14-day period, but to take them out to eat and to clean them with a soft-bristle toothbrush and warm water. Patients had alginate impressions taken of their teeth; the impressions were poured in mounting stone, and clear Essix C+ retainers (Dentsply Sirona, Charlotte, NC, USA) were fabricated in the casts at T0 (day 1). The Essix retainers were composed of a 0.020” (0.5 mm) clear vacuum-forming material. Samples were collected at T0 (day 1), T1 (day 7), and T2 (day 14). The unstimulated saliva and plaque samples were collected from the retainers at day 7 and day 14. It was assumed that at time 0, the retainer was sterile. The alginate impressions, Essix fabrication and sampling were performed by one individual. 

### 2.1. DNA Extraction Library Preparation and Illumina MiSeq Sequencing

We extracted DNA from saliva and plaque was collected from the clear retainer using the QiaAmp DNA kit (Qiagen, Hilden, Germany); we quantified it with a Nanodrop ND-1000 Spectrophotometer (Industriestrasse, Sursee, Switzerland) at a wavelength of 260 nm. Next generation sequencing library preparations and Illumina MiSeq sequencing were conducted at Rutgers Genomic Center, NJMS, Newark, NJ, USA. DNA samples were quantified using a Qubit 2.0 Fluorometer (Invitrogen, Carlsbad, CA, USA). Genomic DNA (20 ng) was used to generate amplicons using an Illumina compatible library preparation kit (NJMS Genomic Center, Newark, NJ, USA). We selected V3-4 hypervariable regions of prokaryotic 16S rDNA for generating amplicons and the following taxonomy analysis. The V3 and V4 regions were amplified using forward primers containing the sequence “5′-TCGTCGGCAGCG TCAGATGTGTATAAGAGACAGCCTACGGGNGGCWGCAG” and reverse primers containing the sequence “5′-GTCTCGTGGGCTCGGAGATGTGTATAAGAGACAGGACTACHVGGGTATCTAATC C”. After the 1st round, the PCR products were used as templates for the 2nd round of amplification utilizing Illumina NexteraXT-indexed primers (Illumina Way, San Diego, CA, USA). Pooled DNA libraries were loaded on an Illumina MiSeq instrument according to the manufacturer’s instructions (Illumina, San Diego, CA, USA). Sequencing was performed using a 2 × 300 paired end (PE) configuration; Image analysis and base calling were conducted by the MiSeq Control Software (MCS) embedded in the MiSeq instrument (Illumina, San Diego, CA, USA).

### 2.2. Data Analysis of Bacterial 16S rRNA Gene Sequencing

Raw metabarcoding reads were assessed for quality control (FASTQC v0.11.9) and trimmed for quality and adapter contaminants (cutadapt v3.2). Amplicon sequence variants (ASVs) were identified and assigned taxonomic classifications (DADA2 v1.20.0) based on the SILVA rRNA database (build 138). Sample rarefaction, alpha diversity calculations, and beta diversity calculations were performed (QIIME2 v2021.2.0). Raw ASV counts normalization and differential ASV expression group cross-comparisons were calculated (DESeq2 v1.26.0) with significance thresholds defined as FDR-adjusted *p* < 0.05. 

### 2.3. Microbiological Evaluation

We collected the saliva and plaque on the retainer as described in the above-mentioned method. The retainer was washed with phosphate buffered saline (PBS; ThermoFisher Sci, Waltham, MA, USA) to remove the non-adhered bacteria prior to vortexing to remove the biofilm from the retainer. The unstimulated salivary bacteria and plaque collected from the retainer surface were serially diluted in the PBS; then, we plated them on a blood agar plate and incubated them in an anaerobic chamber at 37 °C for 3 days, followed by the enumeration of colony forming unit (CFUs) of both the saliva and retainer bacteria.

## 3. Results

**Figure 1:**
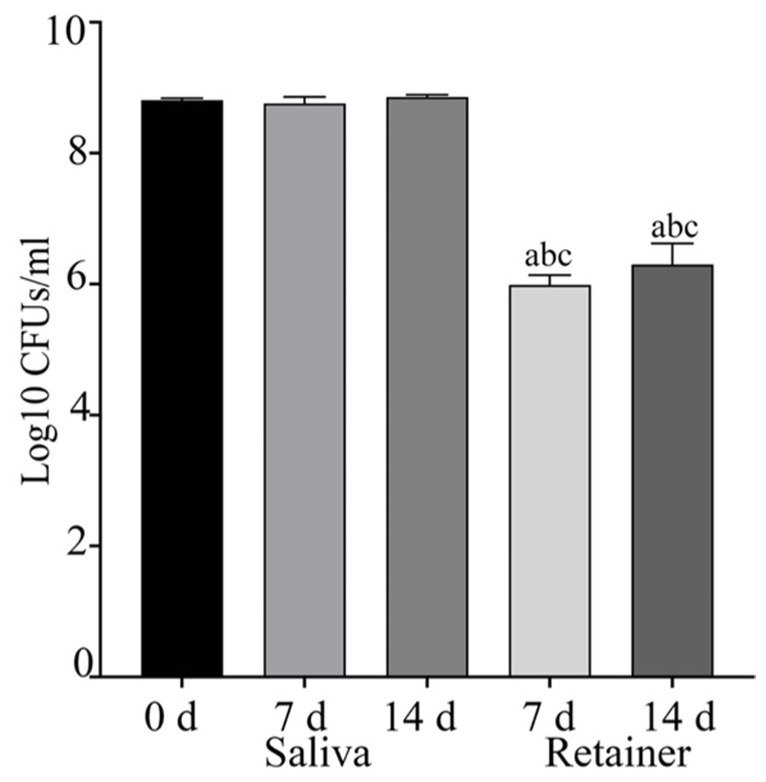
The determined CFUs of salivary bacteria and plaque samples were collected on the retainer surface at 7 and 14 days after retainer treatment. Salivary microbiota at 0, 7 and 14 days showed significantly increased CFUs when compared to the bacteria collected from the retainer surface at 7 and 14 days (<0.0001). a: Compared to salivary bacteria at 0 days; b: compared to salivary bacteria at 7 days, and c: compared to salivary bacteria at 14 days.

To determine the number of CFUs on the clear retainer and salivary bacteria, we collected the bacteria on the retainer and in the saliva at different time point. Figure 1 shows that the saliva at 0, 7 and 14 days; the samples contained significantly more bacteria when compared to the retainer on days 7 and 14 (*p* < 0.001). We also compared the retainer at 7 and 14 days after treatment, and they showed significantly increased bacterial CFUs at 14 days when compared to saliva on day 7 (*p* < 0.0263) (Figure 1).

### 3.1. Microbiome Diversity and Richness of Saliva and Retainer

**Figure 2:**
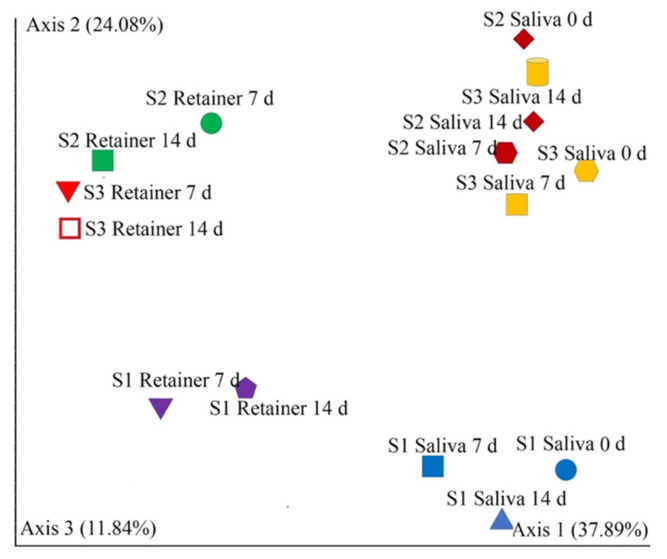
Beta diversity of the oral microbiota following placement of retainer. Beta diversity Jaccard index, based on Principal Coordinates Analysis (PCoA) of all samples. There were no significant differences in the composition and structure of oral flora between the saliva and retainer. The sample spread was statistically different based on Permutational Analysis of Variance (PERMANOVA) (*p* < 0.05). The saliva groups are clustered in the right part of the main horizontal axis. The retainer group is clustered in the left part of the main horizontal axis. Abbreviations: round, saliva 7 d; cylinder, saliva 14 d; cone, retainer 7 d; diamond, retainer 14 d.

Alpha diversity, a measure of microbial community evenness and richness in each sample, was calculated and compared between salivary bacteria and plaque bacteria collected from the retainers at 7 and 14 days. Chao1 and Abundance-based coverage estimators (ACE) were measured to evaluate the richness of the salivary microbiota, while the Shannon index was also used to assess the diversity of the salivary and retainer microbiota. There were no significant changes in alpha diversity across all metrics calculated at 7 and 14 days of retainer treatment (Table 1). Beta diversity, a measure of the variation in microbial communities between saliva and bacteria collected from the retainer at 7 and 14 days, was calculated and compared using Principal Coordinates Analysis (PCoA) based on the Jaccard index. The combined analysis of the beta diversity of the bacteria collected from the retainer and salivary bacteria showed that they were different from each other. We did not observe any significant differences in the composition and structure of the microbiota within the saliva and on the retainer, as represented in Figure 2. 

Comparison of the flora alpha diversity indices. There were no significant differences among the groups. Ordinary one-way ANOVA or Kruskal–Wallis tests were used for the statistical analysis among the different groups. Data are presented as mean ± SEM.

### 3.2. Compared Salivary and Retainer Abundance and Bacterial Composition

**Figure 3:**
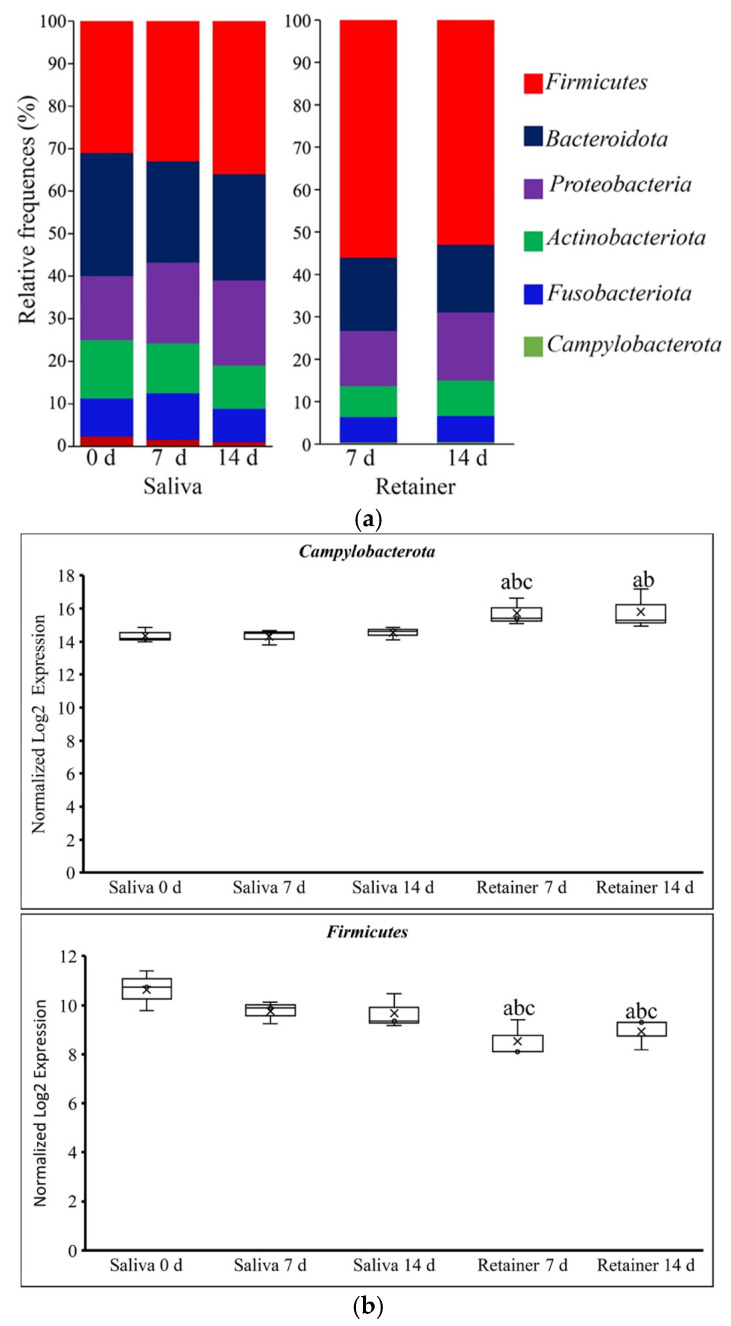
Analyses of the changes in microbial structure at the phylum level. (**a**) The distributions of predominant oral microbial taxa (relative frequency) at the phylum level in the saliva and retainer, with each color representing one phylum. (**b**) Comparison of oral microbiota normalized log2 fold changes at the phylum level in saliva and retainer after 7 and 14 days. a: Compared to salivary bacteria at 0 days. b: Compared to salivary bacteria at 7 days. c: Compared to salivary bacteria at 14 days.

We compared the abundance and microbial distributions at the phylum and genus levels to further study the changes in the microbial community’s structure. At the phylum level, the relative frequencies of the top six phyla are illustrated in Figure 3a, showing that the predominant phyla were *Actinobacteria*, *Fusobacteria*, *Firmicutes*, *Bacteroidetes*, *Proteobacteria*, and *Campylobacterota*, both in saliva and on the clear retainers. As shown in Figure 3b, when we compared the relative abundances of different phyla between the saliva and retainer, the *Firmicutes* were significantly increased 1.33-fold (*p* = 0.0021) at 7 days and 1.5-fold (*p* = 0.0063) at 14 days after the retainer was applied, when compared to the saliva at day 0. When we compared the saliva at 7 days with the retainer at 7 days, the bacteria were significantly increased in the retainer by 1.26-fold (0.0193). Similarly, 1.34-fold increases in retainer bacteria were found when compared to the saliva at 14 days. In contrast, the *Campylobacterota* level was 1.80-fold (*p* = 0.0008) and 1.5-fold (*p* = 0.0051) lower in the retainer at 7 and 14 days, respectively, when compared to the saliva at day 0. The other phyla were equally represented in both the saliva and on the clear retainer. Most of the bacteria found in the saliva were also found on the retainer throughout the 14-day period.

### 3.3. Comparing the Fold Changes in Abundance of Bacteria in Saliva at 0 Days and Retainer at 7 Days

**Figure 4:**
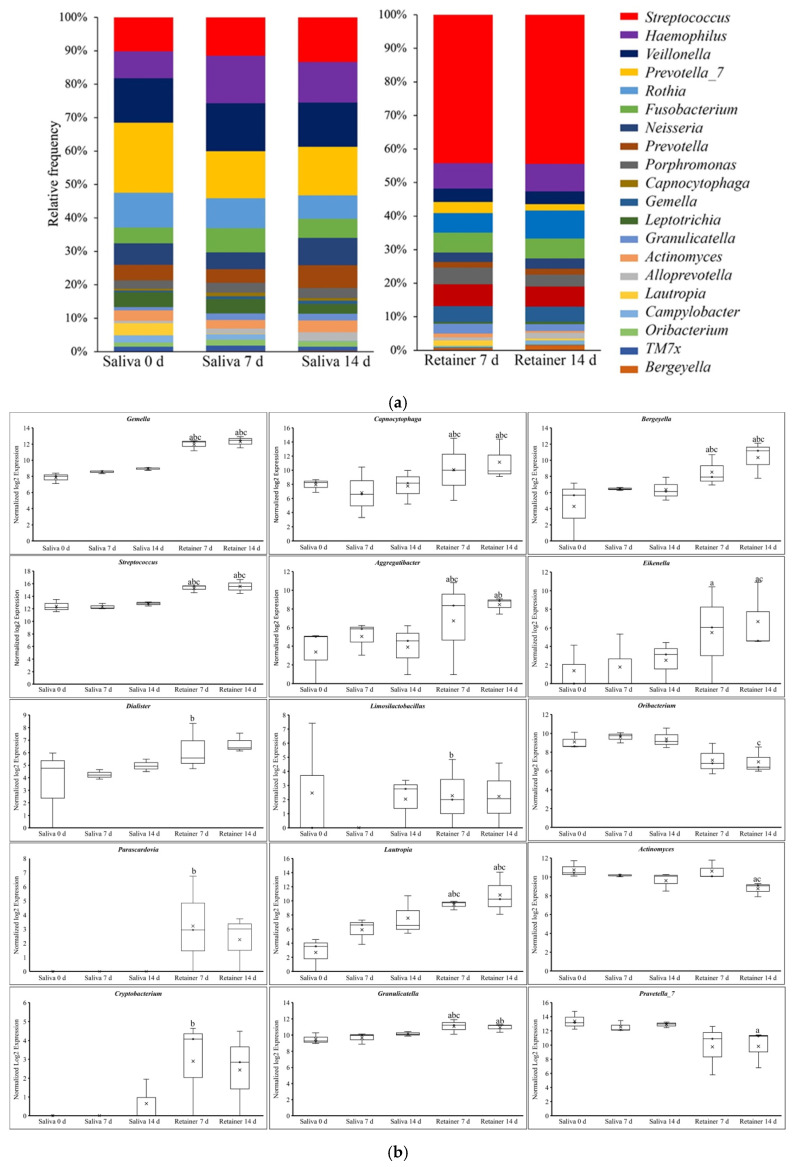
Analyses of the changes in microbial structure at the genus level. (**a**) The distributions of predominant oral microbial taxa (relative frequency) at the genus level in saliva and retainer, with each color representing one genus. (**b**) Comparison of oral microbiota normalized log2 fold changes at the genus level in saliva and clear retainer after 7 and 14 days. a: Compared to salivary bacteria at 0 days. b: Compared to salivary bacteria at 7 days. c: Compared to salivary bacteria at 14 days.

We compared the abundance and microbial distributions at the genus level to further study the changes in the microbial community’s structure. At the genus level, the bar plot in Figure 4a demonstrates the relative abundance of the top genera. When we compared the fold changes in different genera between saliva at 0 days and the retainer at 7 days, the following genera were significantly increased in the retainer when compared to saliva: *Gemella* (3.93-fold; *p* = 0.0000), *Streptococcus* (2.61-fold; *p* = 0.001) *Capnocytophaga* (3.90-fold; *p* = 0.009), *Bergeyella* (2.90-fold; *p* = 0.0159), *Granulicatella* (1.60-fold; *p* = 0.02081), *Lautropia* (4.80-fold; *p* = 0.0004), Eikenella (3.51-fold; *p* = 0.0527), *Aggregatibacter* (3.8-fold; *p* = 0.0160), *Actinobacillus* 3.50-fold; *p* = 0.0157) and decreased *Campylobacter* (1.90-fold; *p*= 0.0160). Similar trends were observed between saliva at day 0 and the retainer at 14 days (Figure 4b). 

### 3.4. Comparing the Fold Changes of Abundance of Bacteria in Saliva at 0 Days and Retainer at 14 Days

When we compared the relative frequencies of different genera between saliva at 0 days and the retainer at 7 days, the following genera were significantly increased in the retainer when compared to the saliva *Gemella:* (4.3-fold; *p* = 0.0000), *Streptococcus* (3.10-fold; *p* = 0.0000) *Capnocytophaga* (3.90-fold; *p* = 0.0100), *Bergeyella* (4.5-fold; *p* = 0.0001), *Granulicatella* (1.40-fold; *p* = 0.0509) *Lautropia* (7.4-fold; *p* = 0.0000), Eikenella (3.90-fold; *p* = 0.0372) and *Actinobacillus* (2.90-fold; *p* = 0.02979); the following were decreased: *Actinomyces* (2.00-fold; *p* = 0.01273), *Prevetella-7* (2.61-fold; *p* = 0.02824) and *Aggregatibacter* (3.1-fold; *p* = 0.04754) (Figure 4b). 

### 3.5. Comparing the Fold Changes of Abundance of Bacteria in Saliva and Retainer at 7 Days

When we compared the relative frequency of different genera between saliva and the retainer at 7 days, the following genera were significantly increased in the clear retainer when compared to saliva: *Gemella* (3.4-fold; *p* = 0.000), *Streptococcus* (2.87-fold; *p* = 0.0002) *Dialister* (2.62-fold; *p* = 0.0080), *Parascardovia* (4.23-fold; *p*= 0.0236), *Cryptobacterium* (3.40-fold; *p* = 0.0377), *Capnocytophaga* (3.28-fold; *p* = 0.0303), *Bergeyella* (2.64-fold; *p* = 0.0243), *Granulicatella* (1.44-fold; *p* = 0.0358), *Limosilactobacillus* (2.33-fold; *p* = 0.0424), *Lautropia* (2.63-fold; *p* = 0.04663), and *Aggregatibacter* (3.12-fold; *p* = 0.04306) (Figure 4b). 

### 3.6. Comparing the Fold Changes of Abundance of Bacteria in Saliva and Retainer at 14 Days

Similar trends were also observed when we compared the expressions of different genera between saliva and the clear retainer at 14 days; the following genera were significantly increased: *Gemella* (3.40-fold; *p* = 0.000), *Streptococcus* (2.86-fold; *p* = 0.0000), *Bergeyella* (3.93-fold; *p* = 0.0008), *Capnocytophaga* (3.35-fold; *p* = 0.0263), *Lautropia* (2.97-fold; *p* = 0.03031), *Eikenella* (3.70-fold; *p* = 0.0422). The following were decreased: *Actinomyces* (1.92-fold; *p* = 0.0150) and *Oribacterium* (2.1-fold; *p* = 0.0376) (Figure 4b). Several genera at 7 and 14 days were identified in the saliva only, and not on the clear retainer, whereas some were found only on the retainer, and not in the saliva (Figure 4b).

After 7 days of retainer treatment, Cutibacterium, Bifidobacterium, Campylobacter, Bulleidia, Dubosiella, Mogibacterium, Shuttleworthia, Bradyrhizobium, Sphingomonas, Neisseria, and Haemophilus were found in the saliva and not on the retainer, whereas Slackia, Prevotella, Tannerella, UCG8, Solobacterium, Isobaculum, Lactobacillus UCG11, Veillonella, Propionigenium and Leptotrichia, were absent in the saliva and found on the retainer. Similarly, after 14 days of retainer treatment, Tannerella, Bulleidia, Lactobacillus, Lactococcus, UCG11, Mogibacterium and Bradyrhizobium were absent in the saliva and found on the retainer, whereas Slackia, Dubosiella, Mycoplasma, Butyrivibrio and Streptobacillus were found in the saliva and absent on the retainer. 

## 4. Discussions

Our study aimed to identify how the microbiome changes on a common thermoplastic material used as a clear retainer and in saliva over a 14-day period. We found that the microbial community in the saliva and on the clear retainer changed regularly throughout the 14-day period of usage of the clear retainer. The microbial community in the saliva was different between day 7 and day 14, with the abundance of some genera significantly changing from day 7 to 14. The study herein demonstrates some bacterial taxa that were found on the clear retainer at 7 or 14 days, and some of the bacteria were present only in the saliva, while some were not present in the saliva, which is consistent with a study performed by Yan et al. [16]. In, addition, many genera were found on the retainer only, and were absent from the saliva [16]. It has been reported that bacteria found in the saliva but not on the retainer, or on the retainer but not in the saliva, could be explained by the fact that the retainer encloses the teeth, keeping them in a closed environment. When traditional orthodontic brackets are used, they are constantly exposed to the saliva and its bacterial composition [16]. 

The common pathogenic bacteria that inhabit the oral cavity were more frequently found in both the saliva and on the retainer at 7 and 14 days. These bacteria have been shown in the literature to be associated with caries formation, enamel decalcification, gingivitis, and periodontal disease. *Streptococcus* spp. of the *Firmicutes* phylum was found in the highest numbers on the clear retainer at both 7 and 14 days. These data are consistent with the results found by Yan et al., whose study looked at the microbiome on the inner surface of a clear aligner from 0 to 24 h, demonstrating that the abundance of *Firmicutes* increases significantly in the first 24 h of aligner therapy. A systematic review by Lucchese et al. additionally found an increase in *S. mutans* on removable appliances as well [21]. This is significant because it suggests that the clear aligner can harbor *S. mutans* and increase the caries risk for the wearer. In our study, *Granulicatella* was increased approximately 2 log at day 7 when compared to saliva. It has been reported that the genus *Granulicatella* was significantly decreased in the saliva [22], but another report detected *Granulicatella elegans* significantly more highly in plaque in a white-spot lesion group of orthodontic patients [5]. *Parascardovia*, which is most frequently isolated from dental caries, was significantly increased in the retainer when compared to saliva at 7 days. *Gemella* is a functionally anaerobic, Gram-positive cocci causing infective endocarditis, and bacteremia was also significantly increased in the clear retainer at both time points. Similarly, to the results found by Yan et al., *Actinomyces* decreased with the increase in retainer usage. The authors concluded that this may be because there was a short follow-up time of only 24 h in their study. However, this was also noted in our study up to 14 days. This may be because this is not a suitable environment for *Actinomyces*. *Actinomyces* have given rise to variable results in the literature when considering orthodontic appliances. In some situations, they have been shown to increase in plaque and saliva after the initiation of orthodontic treatment, and in other cases they increase once the orthodontic appliances are removed. In our study, the numbers of *Actinomyces* decreased with the increase in retainer usage. This shows that the development of *Actinomyces* depends on the environment, and can be influenced by many different factors [22].

Kado et al. performed a study in which supragingival plaque and saliva samples were taken from individuals undergoing fixed orthodontic therapy at 0 and 6 months after treatment, and at appliance removal. They found that the predominant bacteria in both the plaque and the saliva were *Proteobacteria*, *Firmicutes*, *Bacteroidota*, *Fusobacteriota*, and *Actinobacteriota*. The most predominant phylum at 0 and 6 months was *Proteobacteria*. From 0 months to the removal of the orthodontic appliances, the number of *Firmicutes* decreased. *Proteobacteria* increased from 0 to 6 months, but decreased to lower than pre-orthodontics levels at the time of appliance removal. *Bacteroidota* and *Fusobacteriota* increased from 0 months to appliance removal, and *Actinobacteriota* decreased from 0 to 6 months after treatment and then increased towards appliance removal. TM7 and Spirochetes were also found at the beginning and at appliance removal, but in smaller numbers than other bacteria. Our study also found *Firmicutes*, *Bacteroidota*, *Proteobacteria*, *Actinobacteriota*, and *Fusobacteriota* in the highest frequencies in the samples, which is consistent with the results of Kado et al. [22]. In our study, Spirochetes were also found in lower numbers in both the saliva and on the clear retainer. Our study found the same five most common bacteria as Kado et al. [22]. This indicates that the bacteria found at 7 and 14 days persisted at day 60, and were still present at retainer removal. Increasing retainer usage could be detrimental to the host in terms of increased caries risk and increased chance of periodontal destruction. Yan et al. found a significant increase in *Actinobacteria*, as well, from 0 to 24 h, and a decrease in *Proteobacteria* from 0 to 24 h. Our results also show that the *Actinobacteria* remained constant from 7 to 14 days on both the retainer and in the saliva but was slightly higher in the saliva than on the clear retainer. *Actinobacteria* are consistent members of the environmental microbiome, and have been shown to significantly decrease in number during fixed orthodontic therapy [22]. *Campylobacter* was found in more significant numbers in the saliva than on the clear retainer. Previous studies by Kado et al. reported that *Campylobacter* increased in numbers in saliva samples, as well, after fixed orthodontic therapy. *Campylobacter* is a facultative anaerobe that increases the pathogenesis of periodontal disease [22]. 

In our study, *Solobacterium*, *Parvimonas* and *Selenomonas* were also found at the genus level in the retainer at 7 and 14 days, as were gingivitis-associated bacteria, which were reported in the supragingival plaque [22,23,24]. *Fusobacterium* and *Tannerella* were found in the retainer biofilm at both 7 and 14 days; these have the pathogenic potential to cause periodontitis. *F. nucleatum* forms aggregates with other suspected pathogens in periodontal disease, and thus acts as a bridge between early and late colonizers on the tooth surface [25,26]. Based on the previous results and our study, the retainer sample showed an increase in the genus *Tannerella* in the oral cavity after the placement of the orthodontic appliances. 

From day 7 to day 14, the diversity of bacteria found in the saliva only significantly decreased. At day 7, 12 genera were identified in the saliva that were not found on the retainer, and at 14 days, only 5 genera were found in the saliva that were absent from the retainer. Of these five genera, only three were the same as those found at 7 days. Camelo-Castillo et al. [27] reported that, as the diversity declines, microbial communities become less healthy and less stable. This is consistent with the results found in this study—that as the retainer usage increased from 7 to 14 days, the diversity of the microbial communities declined. This shows that there a poorer oral microbial environment develops with the increased duration of retainer usage. This also indicates that the microbial environment of the saliva can be influenced by the use of a clear retainer, which is contrary to the results found by Zhao et al. [28]. The alpha diversity results indicate microbial richness and uniformity in each sample, which indicates the stability and health of the microecological environment [16]. The beta diversity results indicate differences in the microbial diversity amongst the different groups. In this study, the community shifted in the saliva and on the clear retainer from day 7 to day 14. The abundance of some species decreased, while others increased. There were also differences in the microbial community. It was seen that the duration of retainer usage affects the microbial diversity on the clear retainer. The duration of retainer usage was found to affect the microbial development on the inner surface of the clear retainer. There are several features of the mouth and retainer that can contribute to bacterial accumulation on the inner surface of the retainer, including the microgrooves in the retainer, worn or abraded areas of the retainer, and plaque accumulation on the teeth or at the gingival margin. The microbial changes on the inner surface of the clear retainer are important to determining the best cleaning periods for the retainer. This also enables us to assess the longevity of the retainer before it causes adverse effects in the host.

### 4.1. Limitations to the Study

There were some limitations to this study. While a clear aligner or retainer, or any other foreign object, introduced into the mouth can change the bacterial colonies therein, there are other variables present in the mouth that might have contributed to the bacterial composition in the saliva and on the retainer, which remained constant from days 7 to 14. This includes variables such as the caries risk and tooth anatomy of the patient. All of these variables, which were not controlled for in this study, can influence biofilm formation and the accumulation of bacteria on the clear retainer. Another limitation of the study is the small sample size, which comprised only five subjects. Further studies should be conducted with larger sample sizes. Thirdly, different patients have differing oral hygiene practices. Although the patients were instructed to brush twice a day and floss once a day, each patient shows differing dexterity and competence in oral hygiene practices, and this could influence the composition of bacteria from one subject to the next over a 14-day period. Further studies should employ more strictly managed oral hygiene practices, so that the different retainer and oral environments can be maintained in a similar manner throughout the 14-day period. This is the first study to investigate the composition of bacterial flora on the inner surfaces of clear retainer at 7 and 14 days. Additional studies should be conducted to determine the changes in the oral microflora on clear retainer from 0 to 14 days, instead of focusing mainly on the change from 7 to 14 days. Future studies can be conducted to see if teeth develop the same biofilm as the retainer, and also look at different practices or products that can be used in vivo to eliminate the biofilm that develops on the clear retainer. While many bacteria were found in both the saliva and the retainer at 14 days, many changes still occurred in the bacterial compositions found in the saliva and the retainer from 7 to 14 days, which could be due to the enclosing nature of clear retainer. The bacteria that remained constant from 7 to 14 days included many pathogenic types associated with gingivitis, dental caries, periodontitis, and enamel demineralization. 

### 4.2. Significance of Study

There are several aspects of the mouth and retainer that can contribute to bacterial accumulation on the inner surface of clear retainer, including microgrooves in the retainer, worn or abraded areas of the retainer, and plaque accumulation on the teeth or at the gingival margin. Our study found *Firmicutes*, *Bacteroidota*, *Proteobacteria*, *Actinobacteriota*, and *Fusobacteriota* at the highest frequencies in the samples collected. These bacteria colonized early, and have been found in other studies to still be present up to the end of orthodontic treatment. These early colonizers can cause oral dysbiosis, and increase the caries risk and risk of periodontal disease, in patients undergoing long-term orthodontic treatment. This dysbiosis is expected to be reversed after the removal of the clear retainer. The microbial changes on the inner surfaces of clear retainer is important to understand so that we can determine the best cleaning periods for the retainer. This will also allow us to assess the longevity of the retainer, in relation to its potential adverse effects for the host. Orthodontists should monitor the periodontal conditions of their patients from the beginning to the end of treatment, regardless of the age or health of the patient. Guidelines on the cleaning frequency of clear retainer must be established to ensure proper oral care instructions are available for patients and clinicians. The microbial analysis in this study can offer insights into the maximum usage period of a clear retainer before it causes potential adverse effects in the host.

## 5. Conclusions

The microbial diversity was seen to decrease from 7 to 14 days, and at 14 days, both the saliva and clear retainer were inhabited by many bacteria associated with dental caries, gingivitis, periodontitis, and enamel demineralization in the oral cavity. The development of plaque biofilm is time-dependent, and there were variations from 7 to 14 days; the biofilm increased at 14 days, becoming more pathogenic and showing greater diversity.

## Figures and Tables

**Table 1:** Alpha diversity comparisons between saliva and retainer at 7 and 14 days.

Alpha Diversity	Saliva 0 d	Saliva 7 d	Saliva 14 d	Retainer 7 d	Retainer 14 d
Shannon	5.28 ± 0.79	5.52 ± 0.29	5.62 ± 0.17	4.56 ± 0.55	4.47 ± 1.27
Observed features	132.67 ± 82.95	156.33 ± 22.6	164.67 ± 15.50	145.33 ± 55.87	146.00 ± 57.17
Faith_pd	12.84 ± 3.10	13.45 ± 1.40	13.70 ± 1.32	12.13 ± 2.55	14.76 ± 2.10
Evenness	0.79 ± 0.01	0.76 ± 0.02	0.76 ± 0.2	0.64 ± 0.05	0.62 ± 0.14

## Data Availability

The datasets used and analyzed during the current study are available from the corresponding author upon request.
